# Dispositional mindfulness and employment status as predictors of resilience in third year nursing students: a quantitative study

**DOI:** 10.1002/nop2.56

**Published:** 2016-06-02

**Authors:** Diane Chamberlain, Allison Williams, David Stanley, Peter Mellor, Wendy Cross, Lesley Siegloff

**Affiliations:** ^1^ Flinders UniversityAdelaideSouth AustraliaAustralia; ^2^ Monash UniversityClaytonVictoriaAustralia; ^3^ Charles Sturt University (CSU)BathurstNew South WalesAustralia

**Keywords:** Combined study, compassion fatigue, quantitative study, resilience, stress

## Abstract

**Background:**

Nursing students will graduate into stressful workplace environments and resilience is an essential acquired ability for surviving the workplace. Few studies have explored the relationship between resilience and the degree of innate dispositional mindfulness, compassion, compassion fatigue and burnout in nursing students, including those who find themselves in the position of needing to work in addition to their academic responsibilities.

**Aim:**

This paper investigates the predictors of resilience, including dispositional mindfulness and employment status of third year nursing students from three Australian universities.

**Design:**

Participants were 240 undergraduate, third year, nursing students. Participants completed a resilience measure (Connor–Davidson Resilience Scale, CD‐RISC), measures of dispositional mindfulness (Cognitive and Affective Mindfulness Scale Revised, CAMS‐R) and professional quality of life (The Professional Quality of Life Scale version 5, PROQOL5), such as compassion satisfaction, compassion fatigue and burnout.

**Method:**

An observational quantitative successive independent samples survey design was employed. A stepwise linear regression was used to evaluate the extent to which predictive variables were related each to resilience.

**Results:**

The predictive model explained 57% of the variance in resilience. Dispositional mindfulness subset acceptance made the strongest contribution, followed by the expectation of a graduate nurse transition programme acceptance, with dispositional mindfulness total score and employment greater than 20 hours per week making the smallest contribution. This was a resilient group of nursing students who rated high with dispositional mindfulness and exhibited hopeful and positive aspirations for obtaining a position in a competitive graduate nurse transition programme after graduation.

## Introduction

Nurses work in highly stressful environments and many are particularly vulnerable to conditions such as burnout, anxiety, depression and secondary traumatic stress (Rees *et al*. 2015). Nursing students will graduate into these environments and resilience is an essential acquired ability for surviving the workplace. Nursing students also face stressors from academic workloads and deadlines. They commonly undertake paid employment above clinical placement commitments when already under stress from the many challenges of dealing with university study (Pulido‐Martos *et al*. 2012).

Resilience is also a desired attribute for nursing students. Understanding the relationship between resilience, coping mechanisms such as dispositional mindfulness, compassion satisfaction and self‐compassion and the negative impacts of the workplace combined with study stress, such as compassion fatigue and burnout are central to the results offered in this study (Cyrulnik 2011, Drury *et al*. 2014, Hegney *et al*. 2014, Phillips *et al*. 2014, Craigie *et al*. 2016).

### Background

Resilience is described as using flexible adaptability in the face of personal, workplace and academic related challenges and is recognized extensively in both individuals and social groups (Pulido‐Martos *et al*. 2012, Drury *et al*. 2014, Hegney *et al*. 2014, Rees *et al*. 2015).

Garcia‐Dia *et al*. define resilience as, ‘the ability of a person to recover, rebound, bounce back, adjust or even thrive following misfortune, change or adversity’ (2013, p. 264). Cyrulnik adds that resilience describes an individual's, ‘ability to succeed, to live and to develop in a positive way despite the stress or adversity that would normally involve the real possibility of a negative outcome’ (2009, p2). Resilience is associated with greater self‐compassion and compassion satisfaction (Neff & McGehee 2010). A growing body of knowledge supports the association of stress resilience, psychological empowerment and academic achievement for students in nursing programmes (Hodges *et al*. 2008, Drury *et al*.^2014^, Galbraith *et al*. 2014, Hegney *et al*. 2014). There is evidence that personal resilience helps buffer the negative impact of stress in intrinsically challenging situations, for example, university students who maintain both academic and work‐related responsibilities.

Mindfulness is defined as, ‘the awareness that emerges through paying attention on purpose, in the present moment and non‐judgmentally to the unfolding of experience moment to moment’ (Kabat‐Zinn 2003, p. 145). Dispositional mindfulness is not the same as mindfulness meditation, where people make a conscious, focused practice of attending to their current state and sensations. Most recent evidence suggests that dispositional mindfulness could also be described in terms of an innate multifaceted construct characterized by different features that include observing, acting with awareness, non‐judging, self‐compassion and non‐reactivity or acceptance (Feldman *et al*. 2007, Chiesa 2013, Jha *et al*. 2014). When dispositional mindfulness is present, individuals experience the world free or unfiltered from elaboration or internalization and therefore their self‐esteem is less likely to be impacted on by positive or negative daily occurrences (Heppner *et al*. 2008). Dispositional mindfulness is also associated with better performance on a wide range of cognitive tasks that have implications for maintaining psychological health. Dispositional mindfulness is therefore conceptualized as a stable state or ‘disposition’ and can be seen as an inherent personality trait, though it can also be taught (Kabat‐Zinn 2003, Feldman *et al*. 2007, Heppner *et al*. 2008, Chiesa 2013).

Recent studies have focussed on mindfulness interventions in nursing students to alleviate stress and promote resilience (Goff 2011, Foureur *et al*. 2013, McGillivray & Pigeon 2015) with Watson *et al*. (2008) suggesting that nursing student's personality and coping inability are related to adverse psychological outcomes. As burnout and compassion fatigue are such a detriment to nurse well‐being and the nursing workforce overall it is essential to uncover if and to what extent student nurses may be suffering from these debilitating affective/cognitive states. There are gaps in understanding the complex relationships between factors affecting resilience and innate qualities such as mindfulness and self‐compassion. Few studies have explored the relationship between resilience and the degree of innate dispositional mindfulness, compassion, compassion fatigue and burnout in nursing students, including those who find themselves in the position of needing to work in addition to their academic responsibilities.

## The study

### Aim

The aim of this study was to observe the predictors of resilience in third year nursing students as a strategy for dealing with or managing study and workplace related stress. Specifically assessed were, innate dispositional mindfulness, professional quality of life and employment during study enrolment to determine if these psychological and workload states were predictive of personal resilience.

### Methodology

This study was an observational quantitative successive independent samples survey design.

### Method

#### Design

The sample used for this study was a subset of participants from the International Collaboration on Workforce Resilience 1(ICWR‐1) which was a larger ongoing study to test a Workplace Resilience Model in both university students and the nursing workforce. A complete description of the methodology and findings from the ICWR‐1 study are available elsewhere (Rees *et al*. 2015). Recruitment and data collection took place from December 2014–June 2015 at different times across the three sites. The invitations sent out by the universities contained a letter of invitation, the Participant Information Sheet and a link to the Qualtrics Survey (Qualtrics Labs Inc.) hosting site. Five successive survey availabilities dependant on class scheduling were offered with an average of a 20% response rate. To ensure reliability of the study, the inclusion criteria for the study required participants to be currently enrolled students at the university. No mindfulness intervention was performed.

#### Participants

Participants were 240 undergraduate nursing students from three Australian Universities, two metropolitan, one regional, who completed the survey measures. As such the majority of participants were female (*n = *219, 89%, Age: mean = 29 sd 10·56). In terms of country of birth, 75% identified as Australia, 16% as Asia or Pacific Islands, 9% India/Europe/Africa.

All participants were volunteers and were provided with a written explanation of the research project and were given a link to complete the questionnaire via a confidential online survey. Five successive survey availabilities were offered with an average of 20% response rate.

#### Resilience variable

##### Resilience measure Connor–Davidson Resilience Scale (CD‐RISC10)

The Connor–Davidson Resilience Scale (Connor & Davidson 2003) was developed as a survey based measure of stress, coping and ability or resilience. Evidence from previous studies in the community (Hegney *et al*. 2007) and nursing populations (Gillespie *et al*. 2009) suggests that this scale is a valid and reliable measure of resilience for a range of normal and clinical populations (Connor *et al*. 2003). The original 25 item scale uses a five point response scale. Higher scores reflect greater resilience. This study used a shorter 10‐item version that assessed aspects of resilience pertaining to a central core resilience construct (Campbell‐Sills & Stein 2007). The abridged CD‐RISC‐10 version reflects the ability to tolerate experiences such as change, personal problems, illness, pressure, failure and painful feeling (item's examples: ‘Able to adapt to change’, ‘Tend to bounce back after illness or hardship’ and ‘Can stay focused under pressure’). In our sample, the CD‐RISC‐10 showed high internal consistency (Cronbach's α  =  0·90).

#### Mindfulness and professional quality of life variables

##### Cognitive and Affective Mindfulness Scale Revised (CAMS‐R)

The 12‐item Cognitive and Affective Mindfulness Scale–Revised (CAMS‐R, Feldman *et al*. 2007) was used to measure individual differences in mindfulness either as a total score or with the four subset domains of mindfulness (attention, present focus, awareness and acceptance/non‐judgment). The scale and its subsets demonstrated acceptable levels of internal consistency in our sample. (Cronbach's α  =  0·79–0·84).

##### The Professional Quality of Life Scale version 5 (PROQOL5)

The *Professional Quality of Life Scale* version 5 (ProQOL5) (Stamm 2010) is a 30‐item self‐report measure of the positive and negative aspects of helping professions. Professionals rate themselves on each of the 30 statements on a five‐point Likert scale (1 = never 5 = very often). The measure yields subscale scores for Compassion Satisfaction, Burnout and Secondary Traumatic Stress. The Compassion Satisfaction subscale measures the extent to which an individual is able to derive pleasure from doing his or her work well or helping others (e.g. I am happy that I chose to do this work). Higher scores on the Compassion Satisfaction scale indicate higher level of functioning and are correlated with self‐compassion (Stamm 2010). The Burnout subscales measure a professional's feelings of hopelessness and difficulties in dealing with doing his or her job effectively (e.g. ‘I feel trapped by my job as a helper’). Higher scores on the Burnout scale indicate higher level of burnout. The Secondary Traumatic Stress subscale measures work‐related, secondary exposure to people who have experienced trauma (e.g. I find it difficult to separate my personal life from my job as a helper). Higher scores on the Secondary Traumatic Stress scale indicate higher level of secondary trauma (Stamm 2010). The current sample yielded the following internal (Cronbach's α) consistency coefficients: Compassion Satisfaction (0·86), Burnout (0·76) and Compassion Fatigue (0·84).

#### Demographic data

Information that described the participants included what programme of study they were enrolled in, past nursing and tertiary qualifications, employment and the amount of hours while studying and if they were expecting to be accepted into a graduate nurse transition programme or internship at the completion of their studies.

#### Ethical approval

Human research ethics approval was sought and secured at each university, Site 1 (HREC reference code: SONM28‐2014), Site 2 (HREC reference code: CF14/2126 – 2014001130) and Site 3 (HREC reference code: 2014/157).

### Analysis

Statistical analyses were performed using STATA 14 (StataCorp, College Station, TX, USA). The data from the three sites were extracted from the total ICWR‐1 data and incomplete data and surveys were excluded leaving a sample of 240 students. All continuous data were deemed normally distributed after skewness and kurtosis analysis. Descriptive data were presented across the three tertiles of resilience (CD‐RISC) scores as high, medium and low categories to describe the distribution of resilience related to the demographic variables. Differences between the three groups were tested using the chi‐square test, Cochrane's Q test or variance analysis according to anova.

Stepwise Linear regression and the ‘margins’ postestimation command to obtain estimated marginal means and associated confidence intervals were used to evaluate the extent to which resilience (CD‐RISC total score) was related to each predictive variable. Using seven selected predictor variables to run a multiple regression, this study needed a minimum sample size of 153 subjects to achieve 95% power and a medium effect size (0·15) at α = 0·05. Predictive variables were tested for multicollinearity and the residuals were tested for normal distribution by means of the Kolmogorov–Smirnov test. An *a priori* alpha of *P < *0·05 was established for this study.

## Results

### Descriptive statistics

Of the 240 students, the greatest proportion (*n = *194, 79%) were studying on campus, were enrolled in a Bachelor of Nursing Program (*n = *230, 94%) and were fulltime students (*n = *207, 85%). Seventy‐five per cent (*n = *183) had been employed in the last 4 weeks prior to the survey and 45% of these (*n = *110), had been employed greater than 20 hours per week. Seventy eight percent (*n = *192) were expecting to be accepted into a graduate nurse transition programme at the completion of their studies. The mean resilience score was high (37 ± 7) and the distributions of the tertiles of resilience were different (*P < *0·001). The mindfulness (CAMS‐R) mean score was different for each category of resilience (F (2, 239) = 66·19, *P < *0·0005, Wilk's Λ = 0·62, partial η^2^ = 0·37), as were the compassion satisfaction mean scores (*F* (2, 239) = 33·5, *P < *0·0005, Wilk's Λ = 0·77, partial η^2^ = 0·22) and the compassion fatigue mean scores (*F* (2, 239) = 27·6, *P < *0·0005, Wilk's Λ = 0·80, partial η^2^ = 0·19). Refer to Table 1 for Demographic Variables stratified by High, Medium and Low Resilience scores.

**Table 1:** Demographic variables stratified by high, medium and low categories of resilience scores

	Resilience groups^b^			*P* value	Total
	Low	Medium	High		
	0–35	36–41	42–50	**0·000**	37 ± 7
Total *n* (%)	89 (37)	80 (32)	71 (29)	0·561	240
Age (in years)	28 ± 9	31 ± 11	30 ± 10	0·104	29 ± 10
Female	81	74	60	0·492	219 (89)
Male	8	6	11	0·763	26 (11)
Single	49	43	41	0·693	137 (56)
Residential status Australia	76	70	67	0·548	217 (87)
Programme of study BN	84	76	65	0·814	230 (94)
Study Full time	69	70	63	0·094	207 (85)
Mode of study on campus	70	60	61	0·469	194 (79)
Tertiary qualifications other than nursing	23	23	23	0·856	86 (35)
Been employed in the last 4 weeks	68	58	62	0·827	183 (75)
Work >20 hours week					
Yes	43	34	31	0·491	110 (45)
No	45	46	40	0·523	134 (55)
Graduate programme^a^					
Yes	54	71	63	**0·000**	192 (78)
No	35	9	8	**0·000**	53 (22)
Burnout score	29 ± 4·9	28 ± 5·6	29 ± 5·2	0·415	29 ± 5
Compassion score	32 ± 6·1	37 ± 4·2	38 ± 6	**0·000**	36 ± 6
Compassion Fatigue score	50 ± 10	44 ± 7·8	39 ± 9·8	**0·000**	45 ± 9
Mindfulness score^c^	28 ± 4·0	31 ± 3·6	36 ± 4·6	**0·000**	32 ± 5

Values are expressed as mean ± sd, or *n* (%). Values are rounded and may not total 100%. Bold values indicate *P* < 0.05.

a Expect to be accepted into a graduate nurse transition programme at the completion of their studies.

b Resilience (CD‐RISC) groups determined by tertiles.

c Mindfulness (CAMS‐R) total score.

BN, Bachelor of Nursing.

Correlations between resilience, dispositional mindfulness, burnout, compassion satisfaction, compassion fatigue, employment > 20 hours per week and expecting to be accepted into a graduate nurse transition programme are summarized in Table 2. Resilience correlated positively with dispositional mindfulness (*r* = 0·644, *P < *0·001) and compassion satisfaction (*r* = 0·494, *P < *0·001) and negatively with compassion fatigue (*r* = −0·472, *P < *0·001). Compassion fatigue correlated positively with burnout (*r* = 0·529, *P < *0·001) and negatively with dispositional mindfulness (*r* = −0·465, *P < *0·001) and compassion satisfaction (*r* = −0·4830, *P < *0·001). Compassion satisfaction correlated with dispositional mindfulness (*r* = 0·376, *P < *0·001) and expecting to be accepted into a graduate nurse transition programme (*r* = 0·321, *P < *0·001). Employment status correlated only weakly with compassion satisfaction.

**Table 2:** Correlations of predictor variables and covariates with resilience as the dependent variable

	Resilience	Compassion fatigue	Compassion	Burnout	Dispositional mindfulness	Employment	Graduate programme^a^
Resilience	1·0000						
Compassion fatigue	−0·4724**	1·0000					
Compassion	0·4942**	−0·4830**	1·0000				
Burnout	−0·0568	0·5293**	−0·1032	1·0000			
Dispositional mindfulness	0·6440**	−0·4653**	0·3760**	−0·0326	1·0000		
Employment	−0·0243	0·0653	−0·1506*	0·1459	0·0080	1·0000	
Graduate programme^a^	0·3201**	−0·2922**	0·3219**	−0·1243	0·2545**	−0·0714	1·0000

*P < 0·5; **P < 0·001.

a Expect to be accepted into a graduate nurse transition programme at the completion of their studies. Employment >20 hours week.

A forward hierarchical linear regression analysis (adjusted for age and gender) was chosen to test the predictive validity for dispositional mindfulness (CAMS‐R), professional quality of life (ProQOL5), employment status and expecting to be accepted into a graduate nurse transition programme at the completion of their studies on the variance in resilience according to the research aim. Variables were entered into the model in a hierarchical manner and retained if *P < *0·2. Preliminary analyses were conducted to ensure no violation of the assumptions of normality, linearity, multicollinearity and homoscedasticity. A summary of the hierarchical regression is presented in Table 3.

**Table 3:** Hierarchical linear regression analysis with dispositional mindfulness, acceptance subscale, compassion satisfaction, compassion fatigue, employment and graduate nurse transition programme acceptance predicting resilience scores

Step	Variable	β	*R* ^2^	∆*R* ^2^	Ω^2^
	Age	0·081^a^	0·0155		
	Gender	–			
1.	DM total score	0·281**	0·4746	0·4591	0·031
	Ms Acceptance	1·37***			0·125
	Ms Attention	–			
	Ms Present Focus	–			
	Ms Awareness	–			
2.	Compassion satisfaction	0·274***	0·5570	0·09	0·083
	Compassion fatigue	−0·065^b^			0·008
	Burnout	–			
3.	Employment	0·924^a^	0·5613	0·0043	0·004
4.	Graduate programme	1·801*	0·5709	0·096	0·017

*P < 0·05; **P < 0·01; ***P < 0·001.

a *P < *0·20.

b *P < *0·10.

–, Removed (dropped) from the model; DM, dispositional mindfulness: Ms, dispositional mindfulness subset (CAMS‐R).

After adjusting for age and gender (removed), dispositional mindfulness (CAMS‐R) and the subscales, acceptance, attention, awareness and present focus were entered at step 1. The present focus covariant was dropped because of between term collinearity. Both attention and present focus were removed from the model leaving the total dispositional mindfulness score and the sub score of acceptance. Model 1 explained a significant increase in the proportion of variance in resilience, *R* ^2^ = 0·47, *F* (2, 238) = 100·1, *P < *0·0001, explaining 45% of the variance in resilience.

Professional quality of life (Pro‐QOL5) total score and its subsets, compassion satisfaction, burnout and compassion fatigue were entered into the model at step 2 and accounted for a significant proportion of the variance in Resilience, *R* ^2^ = 0·56, *F* (1, 239) = 56·1, *P < *0·0001. The burnout subset was dropped from the model. The total variance explained by the model as a whole was 56%, thus explaining 9% of the variance.

At step 3, employment (in the last 4 weeks) was entered into the model and dropped. Then employment (greater than 20 hours per week) was entered into the model *R* ^2^ = 0·56, *F* (6, 239) = 47·31, *P < *0·001. At the last step, expectation of a graduate nurse transition programme acceptance was entered into the model *R* ^2^ = 0·57, *F* (6, 239) = 42·16, *P < *0·0001. The total model explained 57% of the variance in resilience, all covariates were included. This means that employment explained an additional 1·5% of the variance in resilience, when the effects of dispositional mindfulness and professional quality of life were controlled for statistically. All of the observed predictor variables made a statistically significant contribution to the model. In the order of importance, dispositional mindfulness subset acceptance (β = 1·40, Ω^2^ = 0·125) made the strongest contribution, followed by the expectation of a graduate nurse transition programme acceptance (β = 1·76, Ω^2^ = 0·083), with mindfulness total score (β = 0·270, Ω^2^ = 0·031) and employment (β = 0·892, Ω^2^ = 0·004) making the smallest contribution. These beta values represent the unique contribution of each variable, when the overlapping effects of all other variables were controlled for statistically. None of the other demographic variables were predictive of resilience.

**Figure 1:**
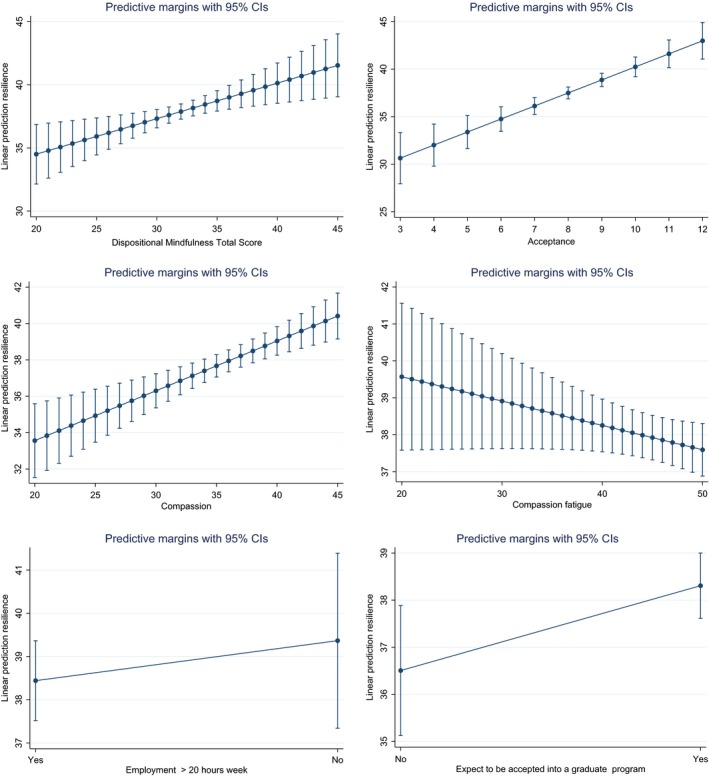
Postestimation Marginal means for Resilience Scores adjusted for age, and gender for independent variables and covariates in the prediction model.

Marginal means were calculated for all predictor variables and presented graphically. Marginal means for continuous variables measures the instantaneous rate of change, which may or may not be close to the effect on the different variables P(Y = 1) of a one unit increase in Resilience. Refer to Figure 1. The marginal effects are the same as the slope coefficients. This is because relationships are linear and do not vary depending on the values of the other variables.

## Discussion

### Resilience

The development of resilience in student nurses is related to their inherent traits, experience and management of stress (Reyes *et al*. 2015). In this study, the cohort resilience scores were high compared with other studies (Neff & McGehee 2010, McGillivray & Pidgeon 2015), but comparable to paramedics and immigrant women studied in Australia (Gayton & Lovell 2012, Loh & Klug 2012). An integrative review conducted on resilience in registered nurses (Hart *et al*. 2014) found that intrapersonal characteristics of hope, self‐efficacy and coping assisted nurses to bounce back and recover from stressful situations. Of the seven studies in Hart *et al*.'s (2014) review, only three reported a mean age of (46·1, 38·4 and 35·4 years) respectively. Suggesting that older nursing students had higher levels of resilience, a trend noted in this study. Gillespie *et al*.'s (2007, 2009) research on Australian operating room nurses found age, experience and education did not contribute to resilience, whereas interpersonal characteristics (alligned with dispositional mindfulness) such as hope, self‐efficacy and coping did.

To maintain resilience requires greater psychological flexibility through the skills of mindfulness and acceptance (Waugh *et al*. 2011). Forty‐five per cent of the variance in resilience was explained by dispositional mindfulness, in particular, the acceptance or non‐judgmental subset of mindfulness. Dispositional mindfulness is an innate characteristic which is associated with salutary coping with stress, enhanced well‐being and with less psychological distress (Roemer *et al*. 2015). As well, lower levels of waking cortisol (the ‘stress hormone’) have been associated with low levels of dispositional mindfulness (Daubenmier *et al*. 2014, Laurent *et al*. 2015). Olson *et al*. (2015) survey of first year US paediatric medical residents found mindfulness and self‐compassion were positively associated with resilience and inversely related with burnout, although gender was not related to resilience. Surprisingly, 40% of medical residents met the criteria for burnout. In this study, only 1% of the cohort met the criteria for burnout (Stamm 2010), but they were also working >20 hours week and were rated in the highest tertile of compassion fatigue and the lowest tertile of both compassion satisfaction and resilience.

In keeping with Hegney *et al*. (2014), Drury *et al*. (2014) and Craigie *et al*. (2016), compassion fatigue and burnout each had a negative relationship with resilience. As well, working more than 20 hours per week had a weak, but evident, relationship with resilience, explaining less than 1% of the variance. As such, the benefits of being employed may be offset by the time constraints and additional stressors imposed as student nurses felt obligated to spend time at work, in competition with study time. Dyrbye *et al*. (2010) reports similar findings in medical students, though no published reports to our knowledge are available regarding nursing students and the employment variables linked to resilience.

A survey of third year nursing students (*n = *217) in China found that self‐efficacy (related to resilience and self‐care) contributed to optimism and problem‐solving and reduced stress (Zhao *et al*. 2014). In addition, Hodges *et al*. (2008) found that new graduate registered nurses in the USA, who are able to effectively problem solve and were goal‐oriented, were more resilient. It is worth noting though, that these findings may be affected by cultural issues.

Expectations of receiving a graduate place following completion of the nursing degree explained 9% of the variance of resilience. A graduate nurse transition programme in Australia is highly sought after and is believed by new graduate registered nurses to provide a solid foundation for practice (Mellor & Greenhill 2014). At the time of the study, nursing students, in particular those from one site saw a downturn in graduate nurse transition programmes severely delaying their career prospects. The students’ positive thinking and emotional tenacity, all features of resilience qualities were demonstrated by 78% of this cohort hopeful of a graduate placement.

The shortage of nurses remains a global issue, where reducing nursing turnover is an economic and quality of patient care imperative (Chachula *et al*. 2015). Challenging, ever changing workplaces, job frustrations, diminished work–life balance and the reality of workplace dissonance reduces resilience and contributes to job stress and turnover of new graduate registered nurses (Hodges *et al*. 2008, Hart *et al*. 2014).

The ability to ‘bounce back’ following adverse circumstances requires personal traits such as hardiness, optimism, hope, self‐efficacy, mindfulness and coping, where student nurses can learn behaviours to cope with stressful work environments before they graduate (Hart *et al*. 2014). Reeve *et al*. (2013) highlight the need for nurse educators and academics to ensure that students develop positive coping strategies or learn to enhance the coping strategies they find successful, to successfully transition into the nursing workforce.

### Limitations

In this study, five successive survey availabilities were offered with an average of a 20% response rate. The students who volunteered for the survey may have been the more resilient of targeted student groups, in keeping with the higher resilience scores in this cohort. Despite this, the sample did provide some insights into resilience in third year nursing students prior to transition into the workforce. As resilience is needed for the nursing workforce, future longitudinal studies could follow a highly resilient student nurse population and investigate the adaption to the nursing workforce.

## Conclusion

The results offered in this study are from an observational successive independent samples survey where 240 undergraduate nursing students from three universities in Australia voluntarily responded. The resilience scores were high overall compared with other similar surveys. The strongest predictors of resilience were dispositional mindfulness and its subset of acceptance. Compassion fatigue and burnout correlated negatively with resilience. Employment greater than 20 hours per week explained only 1% of the variance in resilience. This was a resilient group of nursing students who rated highly with dispositional mindfulness and exhibited hopeful and positive aspirations for obtaining a position in a competitive graduate nurse transition programme after graduation.

## Conflict of interest

There are no conflicts of interest for any of the contributors to this paper.

## Author contributions

All authors have agreed on the final version and meet at least one of the following criteria [recommended by the ICMJE (http://www.icmje.org/recommendations/)]:

- substantial contributions to conception and design, acquisition of data or analysis and interpretation of data;
- drafting the article or revising it critically for important intellectual content.

## References

[nop256-bib-0001] Campbell‐Sills L. & Stein M.B. (2007) Psychometric analysis and refinement of the Connor‐Davidson Resilience Scale (CD‐RISC): validation of a 10‐item measure of resilience. Journal of Traumatic Stress 20(6), 1019–1028. 18157881 10.1002/jts.20271

[nop256-bib-0002] Chachula K.M. , Myrick F. & Yonge O. (2015) Letting go: how newly graduated registered nurses in Western Canada decide to exit the nursing profession. Nurse Education Today 35, 912–918. 25862074 10.1016/j.nedt.2015.02.024

[nop256-bib-0003] Chiesa A. (2013) The difficulty of defining mindfulness: current thought and critical issues. Mindfulness 4(3), 255–268.

[nop256-bib-0004] Connor K.M. & Davidson J.R. (2003) Development of a new resilience scale: the Connor‐Davidson Resilience Scale (CD‐RISC). Depression and Anxiety 18, 76–82. 12964174 10.1002/da.10113

[nop256-bib-0005] Connor K.M. , Davidson J.R.T. & Lee L.‐C. (2003) Spirituality, resilience and anger in survivors of violent trauma: a community survey. Journal of Traumatic Stress 16, 487–494. 14584633 10.1023/A:1025762512279

[nop256-bib-0006] Craigie M. , Osseriran‐Moisson R. , Hemsworth D. , Aoun S. , Francis K. , Brown J. , Hegney D. & Rees C. (2016) The influence of trait‐negative affect and compassion satisfaction on compassion fatigue in Australian nurses. Psychology Trauma: Theory, Research, Practice and Policy 8(1), 88–97. 10.1037/tra0000050 25961866

[nop256-bib-0007] Cyrulnik B. (2011) Resilience: How Your Inner Strength can Set You Free From the Past. Penguin, New York.

[nop256-bib-0008] Daubenmier J. , Hayden D. , Chang V. & Epel E. (2014) It's not what you think, it's how you relate to it: dispositional mindfulness moderates the relationship between psychological distress and the cortisol awakening response. Psychoneuroendocrinology 48, 11–18. 24971591 10.1016/j.psyneuen.2014.05.012 PMC4503930

[nop256-bib-0009] Drury V. , Craige M. , Francis K. , Aoun S. & Hegney D. (2014) Compassion satisfaction, compassion fatigue, anxiety, depression and stress in registered nurses in Australia: study 2 results. Journal of Nursing Management 22(4), 519–531. 24926496 10.1111/jonm.12168

[nop256-bib-0010] Dyrbye L.N. , Power D.V. , Massie F.S. , Eacker A. , Harper W. , Thomas M.R. , Szydlo D.W. , Sloan J.A. & Shanafelt T.D. (2010) Factors associated with resilience to and recovery from burnout: a prospective, multi‐institutional study of US medical students. Medical Education 44(10), 1016–1026. 20880371 10.1111/j.1365-2923.2010.03754.x

[nop256-bib-0011] Feldman G. , Hayes A. , Kumar S. , Greeson J. & Laurenceau J.‐P. (2007) Mindfulness and emotion regulation: the development and initial validation of the Cognitive and Affective Mindfulness Scale‐Revised (CAMS‐R). Journal of Psychopathology and Behavioral Assessment 29(3), 177–190.

[nop256-bib-0012] Foureur M. , Besley K. , Burton G. , Yu N. & Crisp J. (2013) Enhancing the resilience of nurses and midwives: pilot of a mindfulness based program for increased health, sense of coherence and decreased depression, anxiety and stress. Contemporary Nurse 45(1), 114–125. 24099232 10.5172/conu.2013.45.1.114

[nop256-bib-0013] Galbraith N.D. , Brown K.E. & Clifton E. (2014) A survey of student nurses’ attitudes toward help seeking for stress. Nursing Forum 49(3), 171–181. 24392669 10.1111/nuf.12066

[nop256-bib-0014] Garcia‐Dia M.J. , DiNapoli J.M. , Garcia‐Ona L. , Jakubowski R. & O'Flaherty D. (2013) Concept analysis: resilience. Archives of Psychiatric Nursing 27(6), 264–270. 24238005 10.1016/j.apnu.2013.07.003

[nop256-bib-0015] Gayton S.D. & Lovell G.P. (2012) Resilience in ambulance service paramedics and its relationships with well‐being and general health. Traumatology 18(1), 58.

[nop256-bib-0016] Gillespie B.M. , Chaboyer W. , Wallis M. & Grimbeek P. (2007) Resilience in the operating room: developing and testing of a resilience model. Journal of Advanced Nursing 59(6), 757–772. 10.1111/j.1365-2648.2007.04340.x 17608683

[nop256-bib-0017] Gillespie B.M. , Chaboyer W. & Wallis M. (2009) The influence of personal characteristics on the resilience of operating room nurses: a predictor study. International Journal of Nursing Studies 46(7), 968–976. 17915223 10.1016/j.ijnurstu.2007.08.006

[nop256-bib-0018] Goff A.‐M. (2011) Stressors, academic performance and learned resourcefulness in baccalaureate nursing students. International Journal of Nursing Education Scholarship 8(1), 1–20. 10.2202/1548-923X.2114 21291410

[nop256-bib-0019] Hart P.L. , Brannan J.D. & de Chesnay M. (2014) Resilience in nurses: an integrative review. Journal of Nursing Management 22(6), 720–734. 25208943 10.1111/j.1365-2834.2012.01485.x

[nop256-bib-0020] Hegney D. , Builstra E. , Baker P. , Rogers‐Clark C. , Pearce S. , Ross H. , King C. & Watson‐Luke A. (2007) Individual resilience in rural people: a Queensland study, Australia. Rural and Remote Health 7, 620. 17961001

[nop256-bib-0021] Hegney D. , Craige M. , Hemsworth D. , Osseiran‐Mossion R. , Aoun S. , Francis K. & Drury V. (2014) Compassion satisfaction, compassion fatigue, anxiety, depression and stress in registered nurses in Australia: study 1 results. Journal of Nursing Management 22(4), 506–518. 24175955 10.1111/jonm.12160

[nop256-bib-0022] Heppner W.L. , Kernis M.H. , Lakey C.E. , Campbell W.K. , Goldman B.M. , Davis P.J. & Cascio E.V. (2008) Mindfulness as a means of reducing aggressive behaviour: dispositional and situational evidence. Aggressive Behaviour 34, 486–496. 10.1002/ab.20258 18464229

[nop256-bib-0023] Hodges F.H. , Keeley A.C. & Troyan P.J. (2008) Professional resilience in baccalaureate‐prepared acute care nurses: first steps. Nursing Education Perspectives 29(2), 80–89. 18459622 10.1097/00024776-200803000-00008

[nop256-bib-0024] Jha A.P. , Rogers S.L. & Morrison A.B. (2014) Mindfulness Training in High Stress Professions: Strengthening Attention and Resilience. Mindfulness‐Based Treatment Approaches: Clinician's Guide to Evidence Base and Applications, Vol. 347, 2nd edn Academic Press, Elsevier, London, UK.

[nop256-bib-0025] Kabat‐Zinn J. (2003) Mindfulness‐based interventions in context: past, present and future. Clinical Psychology: Science and Practice 10(2), 144–156.

[nop256-bib-0026] Laurent H. , Laurent S. , Nelson B. , Wright D. & De Araujo Sanchez M.‐A. (2015) Dispositional mindfulness moderates the effect of a brief mindfulness induction on physiological stress responses. Mindfulness 6(5), 1192–1200.

[nop256-bib-0027] Loh M.I. & Klug J. (2012) Voices of migrant women: the mediating role of resilience on the relationship between acculturation and psychological distress. The Australian Community Psychologist 24, 59–78.

[nop256-bib-0028] McGillivray C.J. & Pidgeon A.M. (2015) Resilience attributes among university students: a comparative study of psychological distress, sleep disturbances and mindfulness. European Scientific Journal 11(5), 33–48.

[nop256-bib-0029] Mellor P.D. & Greenhill J. (2014) A patient safety focused registered nurse transition to practice program. Contemporary Nurse 47(1–2), 51–60. 25267127 10.1080/10376178.2014.11081906

[nop256-bib-0030] Neff K.D. & McGehee P. (2010) Self‐compassion and psychological resilience among adolescents and young adults. Self and Identity 9(3), 225–240.

[nop256-bib-0031] Olson K. , Kemper K. & Mahan J. (2015) What factors promote resilience and protect against burnout in first‐year pediatric and medicine‐pediatric residents? Journal of Evidence‐Based Complementary and Alternative Medicine 20(3), 192–198. 25694128 10.1177/2156587214568894

[nop256-bib-0032] Phillips C. , Kenny A. , Esterman A. & Smith C. (2014) Does the choice of pre‐registration paid employment impact on graduate nurse transition: an Australian study. Nurse Education Today 34(4), 532–537. 23871152 10.1016/j.nedt.2013.06.024

[nop256-bib-0033] Pulido‐Martos M. , Augusto‐Landa J.M. & Lopez‐Zafra E. (2012) Sources of stress in nursing students: a systematic review of quantitative studies. International Nursing Review 59(1), 15–25.

[nop256-bib-0034] Rees C. , Breen L.J. , Cusack L. & Hegney D. (2015) Understanding individual resilience in the workplace: the international collaboration of workforce resilience model. Frontiers in Psychology 6(73), 1–7. 25698999 10.3389/fpsyg.2015.00073 PMC4316693

[nop256-bib-0035] Reeve K.L. , Shumaker C.J. , Yearwood E.L. , Crowell N.A. & Riley J.B. (2013) Perceived stress and social support in undergraduate nursing students’ educational experiences. Nurse Education Today 33(4), 419–424. 23246284 10.1016/j.nedt.2012.11.009

[nop256-bib-0036] Reyes A.T. , Andrusyszyn M.A. , Iwasiw C. , Forchuk C. & Babenko‐Mould Y. (2015) Resilience in nursing education: an integrative review. Journal of Nursing Education 54(8), 438–444. 26230163 10.3928/01484834-20150717-03

[nop256-bib-0037] Roemer L. , Williston S.K. & Rollins L.G. (2015) Mindfulness and emotion regulation. Current Opinion in Psychology 3, 52–57.

[nop256-bib-0038] Stamm B.H. (2010) The Concise ProQOL Manual, 2nd edn. ProQOL.org, Pocatello, ID.

[nop256-bib-0039] Watson R. , Deary I. , Thompson D. & Li G. (2008) A study of stress and burnout in nursing students in Hong Kong: a questionnaire survey. International Journal of Nursing Studies 45(10), 1534–1542. 18241870 10.1016/j.ijnurstu.2007.11.003

[nop256-bib-0040] Waugh C.E. , Thompson R.J. & Gotlib I.H. (2011) Flexible emotional responsiveness in trait resilience. Emotion 11(5), 1059. 21707168 10.1037/a0021786 PMC3183326

[nop256-bib-0041] Zhao F.F. , Lei X.L. , He W. , Gu Y.H. & Li D.W. (2014) The study of perceived stress, coping strategy and self‐efficacy of Chinese undergraduate nursing students in clinical practice. International Journal of Nursing Practice 21(4), 401–409. 24750234 10.1111/ijn.12273

